# Remedying Contact Dermatitis in Broiler Chickens with Novel Flooring Treatments

**DOI:** 10.3390/ani10101761

**Published:** 2020-09-28

**Authors:** Nathan Freeman, Frank A. M. Tuyttens, Alexa Johnson, Victoria Marshall, An Garmyn, Leonie Jacobs

**Affiliations:** 1 Department of Animal and Poultry Sciences, Virginia Polytechnic Institute and State University, Blacksburg, VA 24060, USA; nathanf1@vt.edu (N.F.); alexaj3@vt.edu (A.J.); victm97@vt.edu (V.M.); 2 Flanders Research Institute for Agriculture, Fisheries and Food (ILVO), 9090 Melle, Belgium; frank.tuyttens@ilvo.vlaanderen.be; 3 Faculty of Veterinary Medicine, Ghent University, 9820 Merelbeke, Belgium; an.garmyn@ugent.be

**Keywords:** animal welfare, footpad dermatitis, poultry, plumage cleanliness, remedial treatment

## Abstract

**Simple Summary:**

Contact dermatitis is an overarching term for inflamed or necrotic lesions after contact with an allergen or irritant. Broiler chickens commonly experience these lesions due to prolonged contact with moisture, feces, and ammonia within litter. This study aimed to find methods to prevent and remedy lesions on broilers’ feet, hocks, and breast. Furthermore, the impact of treatments on plumage cleanliness, gait, and body weight was investigated. We applied novel flooring treatments consisting of plastic slats and disinfectant mats containing povidone-iodine, which we compared to the industry control (used litter) and a positive control (clean litter). Weekly measurements on a sample of birds in each pen showed us the impact of both flooring treatments and age (weeks) on animal welfare outcomes. Contrary to expectations, the novel flooring treatments did not prevent or remedy contact dermatitis. In fact, the positive control, consisting of replacing litter every four days, resulted in the best welfare condition, with limited to no contact dermatitis at week seven of age.

**Abstract:**

Contact dermatitis (footpad dermatitis (FPD), hock burns, and breast dermatitis) is a welfare issue for broiler chickens, causing pain and behavioral restrictions. Once lesions develop, often nothing is done to remedy the issue for the affected flock. Our objective was to evaluate novel flooring treatments at the flock level by providing preventative and remedial treatments against contact dermatitis, plumage soiling, and gait impairment. Broilers (*n* = 546) were housed in 42 pens, with 13 birds/pen. The flooring treatments (four) included used litter (NEG), new pine shavings replaced regularly (POS), a mat filled with 1% povidone-iodine solution (MAT), and the iodine mat placed on a slatted floor (SLAT). Flooring treatments were provided from day one of age (preventative approach; PREV) or day 29 (remedial approach; REM). Contact dermatitis, soiling, gait, and weight were recorded weekly (seven birds/pen). Results showed a treatment effect for all measures, dependent on bird age. Overall, the POS treatment resulted in the best welfare outcomes (FPD, hock burns, and gait). The worst contact dermatitis was found in the MAT and SLAT groups. NEG birds showed little contact dermatitis, opposite to expectations. Weights were lower for PREV-POS in week seven only. The treatments with povidone-iodine were deemed ineffective against contact dermatitis. Access to clean litter prevented and remedied contact dermatitis, and a comparable approach may be commercially feasible.

## 1. Introduction

Contact dermatitis is an inflammation and irritation of the skin due to contact with an irritant or allergen. A common type of contact dermatitis in broiler chickens is footpad dermatitis (FPD), with necrotic lesions on the plantar surface of the central footpad [1]. When these lesions are left untreated, and environmental conditions remain the same or deteriorate, lesions will worsen, eventually encompassing the entire footpad, including the toes [2]. The lesions can be associated with bacterial infections, especially with *Staphylococcus aureus* and *Escherichia coli* (*E. coli*) that can be present in the litter and on the skin [3]. FPD is a common condition found in commercial broiler chickens. Approximately 50% of heavy broilers (3.6 to 3.8 kg) reared on commercial farms in the Southeastern United States (US) present with some degree of FPD [4]. Furthermore, studies in Europe showed FPD in 58% of the assessed commercial broilers [5]. Besides on the feet, similar types of contact dermatitis can occur on hocks (hock burns) and the abdomen (breast dermatitis) [6].

The most important risk factor for the development of FPD is the litter condition [7]. The litter moisture and ammonia concentration from built-up fecal material can burn and weaken the dermis of the footpad [8], with an increased severity of FPD resulting from the prolonged exposure of feet to wet litter. Moisture causes the outer layer of the dermis to soften, posing a risk of microbial contamination, leading to necrosis [9]. Broilers reared on wet litter had 43% of the feet plantar surface affected with FPD compared to 0.2% to 0.3% in broilers reared on dry litter [10]. Risk factors impacting the litter condition and, in turn, FPD include bird sex and size [11], nutrition [12], bedding material [7,13], stocking density [14], and seasonality [6].

Contrary to Europe, where litter is replaced after every flock, the US broiler and broiler breeder industry reuses litter for successive flocks [13,15]. This could be another factor impacting the litter condition and, in turn, poses a risk for contact dermatitis when compared to fresh litter. Chicken feet (paws) can be a highly profitable by-product for the industry, and poor footpad conditions due to FPD reduces the product quality, ultimately resulting in rejections and loss of revenue. The US revenue of chicken feet may vary depending on the trade market, yet was estimated at 270 million USD from export to countries in Asia in 2018 [16]. Moreover, FPD has an overall negative impact on broiler productivity. Untreated FPD lesions can lead to lower body weight gain, feed intake, and water intake, resulting in overall lower carcass weights [17]. FPD lesion scores were positively correlated with condemnation rates and negatively correlated with the live weights and leg meat yields [18]. Therefore, the broiler industry has started to take more interest in preventative and remedial approaches, but there are few feasible treatment options available.

FPD affects bird welfare, especially when lesions are severe and painful [19,20], with behavioral restrictions as a consequence [17]. Chickens have nociceptors in the scale skin of the shanks and feet; therefore, it is considered that broilers feel pain in the presence of severe lesions [19]. Furthermore, FPD can be a gateway for potential bacterial infections and the increased prevalence of lameness [4]. Nearly 47% of heavy broilers (3.6 to 3.8 kg) showed some degree of gait impairment [4]. Broilers with FPD lesions had significantly increased gait impairments in comparison to broilers without lesions [17]. Latency-to-lie test outcomes, indicative of leg weakness, were strongly related to FPD and lameness [21]. Severe gait impairment can be a reason for euthanasia and, thus, an additional loss of revenue. Furthermore, following the EU legislation on fitness for transport, birds with gait impairment would be deemed unfit to travel, resulting in a serious financial loss [22]. Birds affected by FPD and/or lameness can reduce activity, increasing the contact of feet, hocks, and breast with litter material, increasing the susceptibility to abdominal dermatitis and plumage soiling [4,6] and further worsening the risk of feet and hock dermatitis.

Even though a number of risk factors have been identified, the industry rarely takes remedial actions once lesions have developed in a flock. In a number of European countries, a monitoring and penalization system has been developed, where high flock scores at slaughter result in mandatory changes in the management of subsequent flocks, including lowering the stocking density. However, during the production round, little is done to remedy lesions once they have developed. When lesions are small or moderately sized, intervention in terms of replacing the litter could prevent aggravation and result in healing before birds reach the slaughter age [10]. Changing the litter can be unpractical in a commercial system; thus, other remedial interventions need to be studied. Some possible currently applied interventions could include adapting ventilation, thinning the flock, and resolve leaking drinkers. The use of a partially slatted floor was hypothesized to result in good footpad conditions when provided from day one until slaughter [23], although another study found no effect of elevated platform access on FPD scores [24]. The addition of such flooring could potentially remediate lesions, although a remedial approach has not yet been assessed. Additionally, the topical treatment of lesions with an antiseptic could reduce or reverse lesion developments [25]. Yet, in a commercial setting, individual treatment is not feasible. Thus, in addition to litter management, a potential practical antiseptic treatment could be applied. Povidone-iodine is an antiseptic that is bactericidal, fungicidal, tuberculocidal, viricidal, and sporicidal. Iodine can effectively kill yeasts, *Staphylococcus* sp., and *E. coli* [26], with the latter two associated with severe FPD lesions, as birds with FPD may develop more severe lesions due to bacterial infections [3]. Therefore, exposure to the antiseptic could be expected to limit the development of lesions. Thus, the objective of this study was to evaluate novel flooring treatments at the flock level by providing preventative and remedial treatments to prevent or reduce contact dermatitis severity, focusing on FPD, in addition to gait impairment and plumage soiling, in broiler chickens. We hypothesized that (1) access to povidone-iodine would limit the development of contact dermatitis lesions and that (2) access to slatted flooring would limit lesion developments further (by reducing the time spent in direct contact with the litter). In addition, the preventative approach was hypothesized to be optimal, limiting lesion development. The remedial approach was hypothesized to result in the healing of lesions that developed prior to exposure to the antiseptic and slatted flooring.

## 2. Materials and Methods

This experiment was carried out between March and May 2019 and was approved by the Institutional Animal Care and Use Committee (IACUC) of Virginia Tech (protocol 18-246).

### 2.1. Animal Housing

For this experiment, we compared the impact of flooring treatments on contact dermatitis development in a commercial broiler chicken strain. One-day-old male broiler chicks (*n* = 546 Hubbard × Ross) were housed in 42 identical pens of 1.25 m^2^ (10.4 chicks/m^2^ at placement; stocking density of 35.8 kg/m^2^ on day 49) in one climate-controlled research facility until slaughter age of 49 days. Upon arrival from the commercial hatchery (transported for approximately 6 h), chicks were randomly allocated to a pen with 13 birds per pen. Each pen contained a nipple drinker line with three nipples, a feed pan, and pine shavings. A heat lamp was provided during the first 7 days, resulting in continuous lighting during that period. Thereafter, a 18L:6D lighting schedule was applied, with a dark period between 12 a.m. and 6 a.m. A feed flat with starter feed was provided on top of the shavings for the first 7 days to allow for easy access to feed. House temperature was gradually decreased from 32 °C on day 1 to 21 °C on day 49. At the hatchery, birds were vaccinated for Marek’s disease. The birds had ad libitum access to a commercial diet formulated for each life stage (i.e., starter from day 0–16, grower from day 16–27, and finisher from day 27–49). The feed was formulated to contain a high percentage of soybean meal (starter 37% soybean, 57% corn; grower 31.8% soybean, 61% corn; and finisher 29.2% soybean, 63.3% corn) and met nutritional requirements [27]. Soybean meal contains oligosaccharides, stachyose, and raffinose, which are indigestible for monogastrics [28]. Stachyose and raffinose cause a higher concentration of solutes in the lumen of the large intestine, resulting in decreased water absorption. Thus, broilers fed higher levels of soybean meal will have more viscous excreta, aiding in the development of contact dermatitis lesions.

### 2.2. Treatments

The experiment consisted of an incomplete factorial design, with two timing treatments (preventative and remedial) and four flooring treatments. The flooring treatments included a negative control, a positive control, and two novel flooring treatments with disinfectant mats containing a povidone-iodine solution (Table 1). Treatments were randomly allocated over six blocks of pens, resulting in 7 flooring × timing treatments combinations with 6 replicates.

Timing was either preventative (PREV), meaning birds were exposed to the flooring treatments from the start of the experiment (at day 1 of age), or remedial (REM), meaning birds were kept in conditions identical to the negative control (NEG) up until day 29 of age (REM) when they received one of three other flooring treatments (positive control, mat, or mat with slatted flooring).

**Figure 1:**
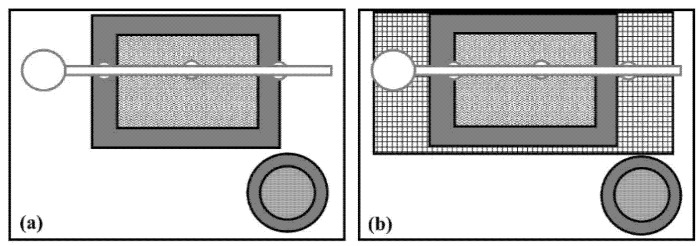
Top view of a mat filled with 1% povidone-iodine solution (MAT) (**a**) and the iodine mat placed on a slatted floor (SLAT) (**b**) pen-based flooring treatments. The pens contained a hanging drinker line (light grey), a metal feeder (dark grey circles), litter (white), a disinfection mat (dark grey rectangle border and patterned fill), and for the SLAT treatment, a plastic slatted floor (rectangle with black and white grid pattern). Illustration is not to scale.

The NEG treatment consisted of housing birds on used litter (19.1% moisture content) that was collected from a previous broiler flock, to model an industry standard. Litter was collected from the experimental pens in the same facility and piled in the center hallway. The used litter was mixed manually and returned to the pens the next day to ensure an equal distribution of litter at a depth of approximately 6 cm. The NEG flooring treatment did not receive a timing treatment. The positive control treatment (POS) was provided as a preventative or remedial treatment, with new pine shavings provided on day 1 (PREV) or day 29 (REM) at a depth of 6 cm, and shavings completely replaced every four days. Pine wood shavings are commonly used as litter for broiler chickens in the United States (US). All treatments received litter at a depth of 6 cm, and PREV-POS is the only treatment group that did not receive used litter at any timepoint. The novel flooring treatment with a disinfectant mat (MAT; Figure 1a consisted of a 60 × 70 cm mat (disinfection mat product number 802010, Agri-Pro Enterprises of Iowa, Inc. Iowa Falls, IA, USA) placed in the back middle of the pen under the drinker line. The rationale for the location of the mats was two-fold: we wanted to ensure all birds in the pen were exposed to the treatment (all birds have to drink), and we wanted to place the mats in a location with a high moisture content. The mat was filled with 3 L of a 1% povidone-iodine solution (diluted with tap water; 050AB Povidone Iodine Solution 10%, Vi-Jon Inc., Saint Louis, MO, USA) and provided on day 1 (PREV) or day 29 (REM). Every four days, the mats were removed from the pen, cleaned, and refilled with the disinfectant solution. The second novel flooring treatment entailed the provision of the MAT treatment (mat plus disinfectant solution), with the additional provision of a black plastic slatted floor (SLAT; Figure 1b) (60 × 120 cm, DURA-SLAT^®^ Black Poultry & Kennel Flooring, Southwest Agri-Plastics Inc. Addison, TX, USA), placed on top of the litter but not elevated from the ground, as commonly done for laying hens and broiler breeders [24]. The mat was placed on top of the slatted floor, and both were placed in the back middle of the pen under the drinker, provided on day 1 (PREV) or day 29 (REM). The slatted flooring was removed as needed to remove excess litter but was not cleaned.

### 2.3. Measurements

At the start of the trial, seven sentinel birds from each pen (*n* = 294 birds) were randomly selected and ring-banded for identification. Only the ring-banded birds were used for measurements each week. Measurements were performed at the start of the trial and then repeated weekly. Measurements included FPD, hock burn, breast dermatitis, plumage soiling, gait, and body weight (Table 2). Each measurement was performed by one observer, eliminating potential interobserver differences, with the exception of body weight, which was recorded by one out of nine people involved. Observers could not be blinded for treatments, as measurements were performed in or near the home pens.

With the exception of weights, all measurements were scored on a tagged visual analog scale (tVAS) of 10 cm labeled with descriptors representing an increasing degree of the welfare concern (FPD, hock burn, breast dermatitis, plumage soiling, and gait/lameness) [32]; thus, higher scores represented a more severe presence of a certain condition. The tVAS ranged from 0 to 100 for all measurements, allowing for precision and sensitivity to the score while having equal or superior interobserver reliability compared to ordinal scales [32]. Ordinal scales as described in Table 2 were transformed to the continuous scale, including the categorical scores as descriptors. For example, gait (0-2 ordinal scale) was scored on a 0–100 continuous scale, where 0 through 33 represented a score 0, 34 through 66 represented a score 1, and 67–100 a score 2. For body weight recording, birds were weighed individually on tabletop scales (8 kg × 0.0001 kg, Adam Equipment Inc, Oxford, CT, USA).

FPD was scored for both feet using the tVAS, tagged with the welfare quality 0 to 4 categorical scale (i.e., score 0 = no lesions, score 1 = small area (≤10%) superficial discoloration, score 2 = large area (≥10%) superficial discoloration, score 3 = deep lesion and ulceration (up to 50%), and score 4 = deep lesion and ulceration (>50%)) [29]. Hock burns were scored for both hocks using the tVAS, tagged with a 0 to 4 categorical scale (i.e., score 0 = no visible discoloration, score 1 = small size (<20%) discoloration, score 2 = small size (20–40%) discoloration, score 3 = moderate size (40–60%) dark discoloration, and score 4 = large size (>60%) dark discoloration) [29]. Breast dermatitis was scored by assessing the breast for inflammation using a tVAS tagged with a 0 to 3 categorical scale (i.e., score 0 = skin without lesion, inflammation, or erythema; score 1 = generalized erythema (up to 25% of ventral body area); score 2 = different degrees of erythema (from 25% to 50% of the ventral body area); and score 3 = large area inflamed (more than 50% of ventral body area), brown spots, or breast blisters) [30]. Soiling of the abdominal feathers was scored using the tVAS, tagged with a 0 to 3 categorical scale (i.e., score 0 = absence, score 1 = light, score 2 = moderate, and score 3 = severe) [31]. Lameness was scored by observing the gait pattern of a bird walking a distance of 1.5 m using a tVAS with a categorical scale from 0 to 2 (i.e., score 0 = no impairment, score 1 = obvious impairment, walk with a clear limp or awkward gait, and score 2 = severe impairment, may shuffle on shanks or hocks with assistance of wings) [31].

In addition to the animal-based welfare measures, litter samples were collected from each pen every week to assess the moisture content. The samples were weighed, dried for 24 h at 100 °C, and then reweighed. The difference between the weights determined the moisture loss, which was expressed as a percentage.

### 2.4. Statistical Analysis

Treatments were randomly allocated over six blocks, with an incomplete factorial design of three flooring treatments × two timing treatments, plus a negative control treatment, resulting in seven treatments (NEG, PREV-MAT, REM-MAT, PREV-SLAT, REM-SLAT, PREV-POS, and REM-POS).

Distribution of residuals were determined through visual assessment of QQ plots for the output variables FPD score (average of two feet), hock burn score (average of two hocks), breast dermatitis score, plumage cleanliness, gait score, and body weight. All outcomes were considered to be normally distributed. Data were analyzed using mixed models with block (*n* = 6) as a random factor and week (*n* = 8) and bird ID (*n* = 294) as repeated factors. The treatments (*n* = 7), week (bird age *n* = 8), and their interactions were included as factors. Tukey-Kramer means separation was used to identify pairwise differences at *p* < 0.05. Due to a human error, birds in 4 pens received incorrect treatments from day 13 through day 16 of age. Data analysis omitting the 4 pens from the dataset revealed that this error did not affect the impact of the independent variables on the dependent variable modeled means. Therefore, authors decided to not omit the 4 pens from the dataset.

## 3. Results

The experimental treatments resulted in differences in FPD, hock burns, breast dermatitis scores, plumage cleanliness, gait, and body weights (Table 3).

### 3.1. FPD

**Figure 2:**
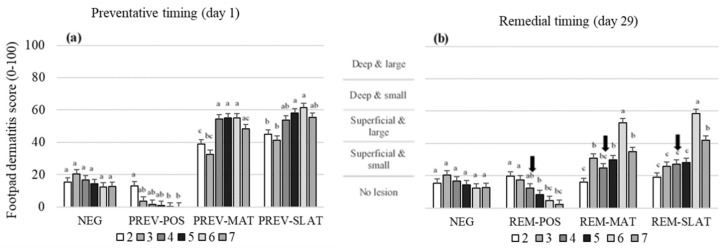
Mean footpad dermatitis scores (raw means ± SEM) of birds that received (**a**) preventative provision of the flooring treatments (PREV) (**b**) or birds that received remedial provision of the flooring treatments (REM): negative control (NEG (presented twice)), clean litter (POS), iodine mat (MAT), or iodine mat with slatted floor (SLAT) during weeks 2 through 7 of age. Scores are on a 0–100 tagged analog scale (tVAS), with increasing scores representing a worsened condition (the “tags” on the VAS are indicated by the horizontal gridlines at each 20-point increase). The arrows in (b) indicate the timing applications of the remedial treatments. Means in a flooring × timing treatment combination with different superscripts (^a–c^) differ significantly at *p* < 0.05.

Treatment, age, and their interaction affected the FPD scores (*p* < 0.001; Table 3). In the preventative approach, PREV-POS showed the best (lowest) FPD scores compared to the other treatments (*p* ≤ 0.002; Figure 2a). The most severe (high) FPD scores were found in PREV-MAT and PREV-SLAT compared to the best (lowest) FPD scores in NEG and PREV-POS (pairwise comparisons between flooring treatments *p* ≤ 0.002; Figure 2a). Similar results were found in the remedial approach (Figure 2b), with REM-MAT and REM-SLAT showing higher FPD scores compared to NEG and REM-POS (pairwise comparisons between flooring treatments *p* < 0.001). In the remedial approach, REM-POS did not differ from the NEG outcomes. Within the PREV-POS and REM-POS treatments, scores decreased with age (*p* < 0.05; Figure 2). Within PREV-MAT, PREV-SLAT, REM-MAT, and REM-SLAT, scores generally increased as the birds aged (*p* < 0.05; Figure 2), whilst scores in the NEG treatment were not affected by age.

### 3.2. Hock Burns

**Figure 3:**
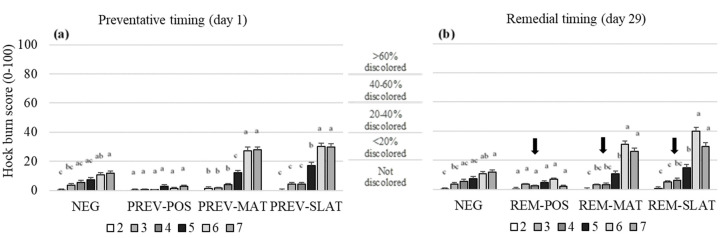
Mean hock dermatitis scores (raw means ± SEM) of birds that received (**a**) preventative provision of the flooring treatments (PREV) (**b**) or birds that received remedial provision of the flooring treatments (REM): negative control (NEG (presented twice)), clean litter (POS), iodine mat (MAT), or iodine mate with slatted floor (SLAT) during weeks 2 through 7 of age. Scores are on a 0–100 tagged analog scale (tVAS), with increasing scores representing a worsened condition (the “tags” on the VAS are indicated by the horizontal gridlines at each 20-point increase). The arrows (b) indicate the timing applications of the remedial treatments. Means in a flooring × timing treatment combination with different superscripts (^a–c^) differ significantly at *p* < 0.05.

Treatment, age, and their interaction affected the hock burn scores (*p* < 0.001; Table 3). In the preventative approach PREV-POS showed the best (lowest) hock burn scores compared to the other treatments (*p* < 0.001; Figure 3a). The most severe (high) hock burn scores were found in PREV-MAT and PREV-SLAT compared to NEG and PREV-POS (pairwise comparisons between flooring treatments *p* < 0.001; Figure 3a). Similar results were found in the remedial approach, with REM-POS showing the lowest scores compared to the other treatment groups (Figure 3b). In addition, REM-MAT and REM-SLAT showed higher hock burn scores compared to NEG and REM-POS (pairwise comparisons between flooring treatments *p* < 0.001; Figure 3b).

Within the PREV and REM-POS treatment groups, the hock burn scores remained low over time, showing no age effect. Within all other treatment groups, the hock burn scores increased with age (*p* < 0.05; Figure 3).

### 3.3. Breast Dermatitis

**Figure 4:**
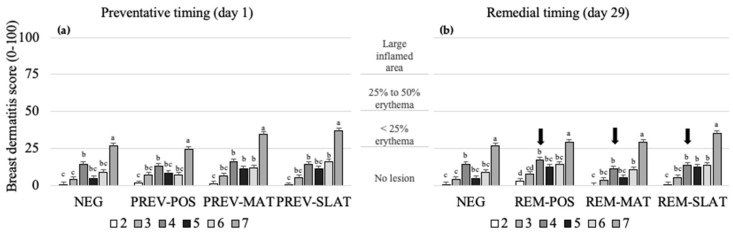
Mean breast dermatitis scores (raw means ± SEM) of birds that received (**a**) preventative provision of the flooring treatments (PREV) (**b**) or birds that received remedial provision of the flooring treatments (REM): negative control (NEG (presented twice)), clean litter (POS), iodine mat (MAT), or iodine mat with slatted floor (SLAT) during weeks 2 through 7 of age. Scores are on a 0–100 tagged analog scale (tVAS), with increasing scores representing a worsened condition (the “tags” on the VAS are indicated by the horizontal gridlines at each 25-point increase). The arrows in (b) indicate the timing application of the remedial treatments. Means in a flooring × timing treatment combination with different superscripts (^a–d^) differ significantly at *p* < 0.05.

Treatment, age, and their interaction affected the breast dermatitis scores (respectively, *p* = 0.008, *p* < 0.001, and *p* = 0.001; Table 3). However, no pairwise differences were found between flooring treatments within the preventative or remedial approaches. Within all treatment groups, the breast dermatitis scores increased with age (*p* < 0.05; Figure 4).

### 3.4. Plumage Cleanliness (Soiling)

**Figure 5:**
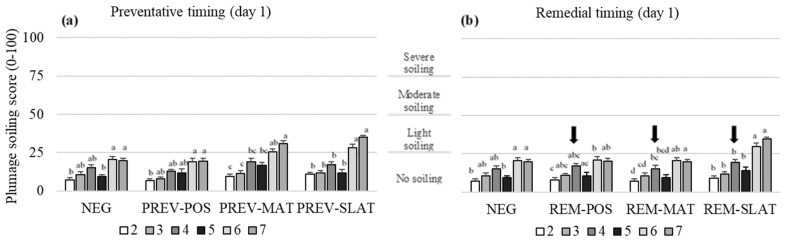
Mean plumage soiling scores (raw means ± SEM) of birds that received (**a**) preventative provision of the flooring treatments (PREV) (**b**) or birds that received remedial provision of the flooring treatments (REM): negative control (NEG (presented twice)), clean litter (POS), iodine mat (MAT), or iodine mat with slatted floor (SLAT) during weeks 2 through 7 of age. Scores are on a 0–100 tagged analog scale (tVAS), with increasing scores representing a worsened condition (the “tags” on the VAS are indicated by the horizontal gridlines at each 25-point increase). The arrows in (b) indicate the timing application of the remedial treatments. Means in a flooring × timing treatment combination with different superscripts (^a–d^) differ significantly at *p* < 0.05.

Treatment, age, and their interaction affected the plumage soiling scores (*p* ≤ 0.001; Table 3). In the preventative application, PREV-POS had lower scores (less soiling) compared to PREV-MAT and PREV-SLAT (*p* ≤ 0.030) but did not differ from the NEG (Figure 5a). In the remedial approach, REM-SLAT showed higher scores (more soiling) compared to the NEG treatment (*p* = 0.024; Figure 5b). No other pairwise differences were found within the preventative or remedial approaches. For all treatments, the plumage soiling scores increased (more soiling) over time (*p* < 0.05; Figure 5).

### 3.5. Gait

**Figure 6:**
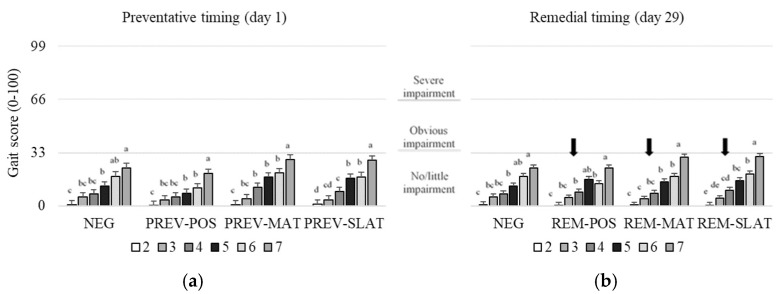
Mean gait scores (raw means ± SEM) of birds that received (**a**) preventative provision of the flooring treatments (PREV) (**b**) or birds that received remedial provision of the flooring treatments (REM): negative control (NEG (presented twice)), clean litter (POS), iodine mat (MAT), or iodine mat with slatted floor (SLAT) during weeks 2 through 7 of age. Scores are on a 0–100 tagged analogue scale (tVAS) with increasing scores representing a worsened condition (the ‘tags’ on the VAS are indicated by the horizontal gridlines at each 33-point increase). The arrows in (**b**) indicate timing application of remedial treatments. Means in a flooring × timing treatment combination with different superscripts (^a–e^) differ significantly at *p* < 0.05.

Treatment, age, and their interaction affected the gait scores (respectively, *p* < 0.001, *p* < 0.001, and *p* = 0.004; Table 3). In the preventative approach, PREV-POS resulted in better (lower) gait scores compared to NEG, PREV-MAT, and PREV-SLAT (*p* ≤ 0.002; Figure 6a). Furthermore, PREV-MAT showed worse (higher) gait scores compared to the NEG (*p* = 0.035; Figure 6a). Within the remedial approach, no pairwise differences between flooring treatments were found (Figure 6b). Within all treatment groups, the gait scores worsened (increased) with age (*p* < 0.05; Figure 6).

### 3.6. Body Weight

**Figure 7:**
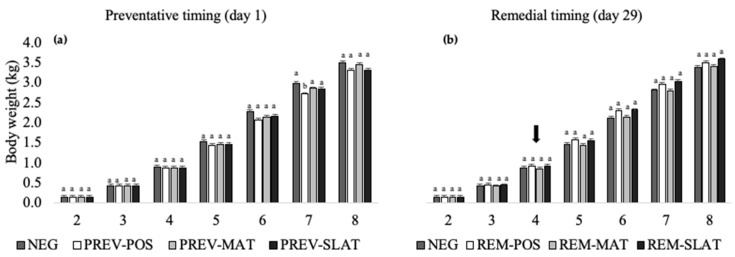
Mean body weights (kg) (raw means ± SEM) of birds that received (**a**) preventative provision of the flooring treatments (PREV) (**b**) or birds that received remedial provision of the flooring treatments (REM): negative control (NEG (presented twice)), clean litter (POS), iodine mat (MAT), or iodine mat with slatted floor (SLAT) during weeks 2 through 8 of age. The arrow in (b) indicates the timing application of the remedial treatments. Means in a flooring × timing treatment combination with different superscripts (^a–b^) differ significantly at *p* < 0.05.

The body weights significantly increased each week in all treatment groups (Figure 7). Although we found an interaction effect between the treatment and week (Table 3), pairwise differences between the groups were not significant within the preventative or remedial applications (Figure 7). Furthermore, limited effects of the flooring treatments were found within a week, resulting in comparable body weights between the treatments, with the exception of week seven, where PREV-POS showed lower weights compared to NEG, PREV-MAT, and PREV-SLAT (Figure 7a).

### 3.7. Litter Moisture

The litter moisture differed over time, with an increasing moisture in the litter as the birds aged from (LS MEAN ± SEM) 9.8% ± 0.9% in week two to 32.6% ± 0.9% in week eight (*p* < 0.001). It tended to differ between treatments (*p* = 0.095), with 22.8% litter moisture in the NEG, 23.4% in REM-POS, 24.3% in REM-MAT, 24.8% in PREV-SLAT, 25.5% in PREV-POS, 25.8% in REM-SLAT, and 27.0% in PREV-MAT (SEM = 1.1%).

## 4. Discussion

Contact dermatitis, and especially FPD, is a prevalent welfare issue for broiler chickens, with potential economic consequences. Common commercial practices do not include any type of treatment of these lesions, which could be costly and labor-intensive if birds would need to be treated individually. Therefore, this study aimed to identify effective preventative and remedial flooring treatments at the flock level to reduce contact dermatitis in broilers. The remedial flooring treatments were compared to preventative applications of the same treatments. To our knowledge, this is the first study assessing an antiseptic (in combination with slatted flooring) as a potential remedy for contact dermatitis, plumage soiling, and lameness.

The flooring treatments applied in this study had an impact on the welfare outcomes, including FPD, hock burns, gait (depending on week of age), plumage cleanliness, and breast dermatitis. Contrary to the hypothesis, however, providing access to an antiseptic solution (povidone-iodine) either from day one or day 29, resulted in more severe FPD lesions as compared to both the negative control (model of the industry standard) and the positive control. The litter moisture hardly differed between the treatments (means ranged from 22% to 27%), so this could not explain the large disparity in lesion scores between the treatment groups. Yet, an inherent part of the MAT and SLAT treatments was exposing birds to a liquid (the antiseptic solution), which could cause increased lesion scores due to softening of the dermis [9], potentially loosening the scales and resulting in an acceleration of lesion development [33]. Furthermore, the mat and slatted floorings took up a large proportion of the pens (25–55% of the floor space). This could contribute to the induction of FPD lesions, as birds were potentially exposed to the moist surface of the mat for prolonged periods of time. Reducing the amount of solution in the mats could have a limited lesion development, yet a priori, we theorized that the liquid would be beneficial, and thus, the mats needed be saturated for the birds to come into contact with the antiseptic solution.

Cengiz et al. [34] reported low FPD severity in broilers kept on plastic wire floors. In contrast, our findings suggest that the slatted floor contributed to FPD lesion developments when exposed from day one but not when exposed from day 29 when we compared MAT to SLAT (Table 3). The slats used in this study are commonly used for broiler breeders, thus were deemed appropriate for the broilers.

Another contributing factor for differences in lesions between the flooring treatments is the chemical and microbial compositions of the litter or on the footpads due to the antiseptic effects of povidone-iodine in SLAT and MAT and due to replacement of the litter in the POS treatment. However, chemical and microbial compositions were not measured in this study. Even though MAT and SLAT resulted in the highest FPD scores compared to the other treatments, the lesions were superficial. A remedial (healing) effect could only be detected if there were a sufficient number of birds that were (badly) affected by dermatitis. It seems at least questionable whether this was the case in this experiment, so the power for detecting a remedying effect may have been low. Indeed, when considering the mean FPD scores for the REM treatments prior to applying the REM treatments in week four, the scores were low (REM-POS 12, REM-MAT 25, and REM-SLAT 27 out of 100), making it less likely for a remedial effect to be detected. Application of the flooring treatments on a commercial scale, where mats and slatted flooring take up a smaller proportion of the space, may be beneficial, yet the current data do not support this.

In-line with previous findings, clean and dry litter effectively prevents [17] and remedies FPD and hock lesions [10,35]. Hock burn scores were consistently low within the POS treatments, the lowest compared to all the other treatment groups. For FPD, both PREV-POS and REM-POS showed a decrease in scores from mean scores of 13 and 19 (out of 100) in week two to 0 and 2 in week seven, which reflected a nearly perfect footpad condition. Both PREV and REM approaches showed a similar pattern for this treatment, where the lesions almost completely healed. The POS treatment showed the greatest reduction in lesion scores compared to any of the other treatments. This treatment shows great promise for application in the broiler chicken industry, albeit with modifications. In the trial, all the litter was replaced in the POS treatments, which is not feasible on a commercial farm. Yet, top-dressing with fresh litter, combined with removing and replacing the worst parts of the litter, has the potential to remedy FPD lesions, although scientific findings are lacking.

The NEG treatment resulted in stable and relatively low FPD scores as the birds aged, which is opposite to the expectation. Exposure to previously used litter was used to model a US commercial standard, and following that exposure, we expected poor footpad quality, as found in the commercial setting [4]. However, the NEG treatment was associated with higher FPD scores compared to the positive control but only when the litter was replaced regularly from day one, as the NEG scores did not differ from REM-POS. The lack of severe lesions in NEG birds was likely due to low litter moisture in the NEG treatment group, similar to findings with turkeys, where dry litter (under 30% moisture) resulted in low FPD scores (<1 on a 0 to 5 scale), regardless of bird age [36]. Another difference compared to the commercial standards was that the used litter came from a single flock, rather than multiple, with the latter approach being linked to more severe FPD lesions with each successive use [13]. In our case, this could have resulted in better-quality litter compared to commercial standards.

For FPD and gait, longer exposure to the flooring treatments (day one compared to day 29; Table 3) resulted in increased severity of these indicators, although the effect was less pronounced for the gait scores. PREV-POS was the best approach to maintain low gait scores compared to all other flooring × timing treatments. For all flooring × timing treatment combinations, the gait scores were relatively low compared to commercial farms [4]; within our study, obvious impairments were only observed in week seven of age (eight birds originating from all treatments; data not shown). In part, this is a result of euthanasia decisions. Severe lameness was a reason to euthanize birds, which impacted the mean gait scores in all the treatments. Out of 294 sentinel birds, 23 died or were euthanized, of which seven were for leg issues when birds were six or seven weeks old (one from a NEG pen, one from a PREV-MAT pen, four from three REM-MAT pens, and one from a REM-POS pen).

Breast dermatitis and plumage soiling scores showed limited responses to the treatments. Compared to birds at a Brazilian slaughter plant [31], the prevalence of breast dermatitis was low in our study. They recorded 72% of birds with a categorical score of 1 or higher (tVAS > 25) at four weeks of age [31], whilst all birds in our study showed a score between 0 and 1 (tVAS < 25) in weeks one through six of age. For all treatments, the mean plumage soiling scores were low, as 0 on the ordinal scale (no soiling) was scored most frequently (71% of the scores; data not shown). These low scores are not in-line with a previous study under comparable stocking density conditions, where 99% of birds showed slight soiling irrespective of the litter treatment [24]. PREV-POS resulted in lower (better) soiling scores compared to PREV-MAT and PREV-SLAT, suggesting that the best strategy to avoid soiling is early access to clean litter that is regularly replaced.

The welfare indicators generally worsened with the bird age. The positive association between FPD, hock burns, breast dermatitis, plumage soiling, and gait with age was in-line with the expectations and findings in the literature: FPD [17,35,37,38], hock burns [17], plumage soiling [17], and gait [17,38].

Bird weights were recorded as an indicator of productivity. The treatments had a minimal effect on the body weights, with the only difference found in week seven of age, where PREV-POS showed lower body weights compared to NEG and the other PREV treatments. In week eight, this difference was no longer observed. Based on observations of a subsample of pens, birds in the POS treatments generally spent more time being active compared to birds in the other flooring × treatment combinations, which could have contributed to the weight difference. The birds were active for 48% (PREV-POS), 43% (REM-POS), 41% (NEG), 39% (REM-MAT), 34% (REM-SLAT), 33% (PREV-MAT), and 32% (PREV-SLAT) of the time they were observed [39]. Due to the limited impact of treatments on weights, we can cautiously conclude that applying these treatments does not negatively impact the productivity, regardless of the lesion scores. This contradicts the previously reported negative relationship of productivity with litter deterioration and FPD at day 37 of age [17].

## 5. Conclusions

This study was the first to evaluate flock-level preventative and remedial approaches to reduce contact dermatitis, focusing on footpad dermatitis in broiler chickens. The exposure to mats with antiseptics, either combined with slatted flooring or on its own, did not prove an appropriate method to prevent or reduce contact dermatitis, plumage soiling, or lameness in the current experiment. The most promising approach to both prevent and remedy these welfare concerns was access to clean litter. In the current study, fresh litter was replaced regularly, which is not feasible in the industry. Yet, removing and replacing litter in certain spots on-farm, top-dressing with fresh litter, and replacing used litter in between flocks seems worth investigating in order to remedy contact dermatitis, especially footpad lesions in commercial flocks.

## Figures and Tables

**Table 1:** Pen-based flooring treatments provided at two different times during production (preventative or remedial). Prior to day 29 treatment, REM pens were kept under NEG conditions with used litter.

Treatment	Timing Treatment (Starting Day)	Flooring Treatment	Description
NEG	N/A (day 1)	Negative control	Birds were housed on used litter from a previous flock from day 1 to day 49, which models the industry standard.
PREV-POS	Preventative (day 1)	Positive control	Birds were provided with new pine shavings at a depth of 6 cm; the shavings were replaced every four days.
REM-POS	Remedial (day 29)		
PREV-MAT	Preventative (day 1)	Mat with iodine solution	Birds were provided a disinfectant mat with 3 L of a 1% povidone-iodine solution; the mats were removed from the pen, cleaned, and refilled with disinfectant solution every four days.
REM-MAT	Remedial (day 29)		
PREV-SLAT	Preventative (day 1)	Mat with iodine solution and slatted floor	Birds were provided a disinfectant mat with 3 L of a 1% povidone-iodine solution and plastic slatted floor; the mats were removed from the pen, cleaned, and refilled with disinfectant solution every four days; excess litter and fecal matter was removed from the slatted floor as needed.

**Table 2:** Measurements (unit) and age of birds on sampling days. All categorical scoring systems were transformed to a continuous score using a tagged visual analog scale.

Measurement		Sampling Day
Contact dermatitis	Footpad dermatitis ^1^ (0–4 score)	11, 18, 25, 32, 39, 46
	Hock burn ^1^ (0–4 score)	11, 18, 25, 32, 39, 46
	Breast dermatitis ^2^ (0–3 score)	9, 16, 23, 30, 37, 44
Plumage soiling ^2^ (0–3 score)		9, 16, 23, 30, 37, 44
Gait ^3^ (0–2 score)		12, 19, 26, 33, 40, 47
Body weight (kg)		1, 8, 15, 22, 29, 36, 43, 49

^1^ Adapted from [29], ^2^ adapted from [30], and ^3^ adapted from [31].

**Table 3:** LS MEANS (and SEM) of the welfare outcomes for all flooring × timing treatment combinations (trt). *p*-values of the main and interactive effects are given for all welfare outcomes.

Welfare Outcome	NEG	PREV-POS	PREV-MAT	PREV-SLAT	REM-POS	REM-MAT	REM-SLAT	SEM	*p*-Value		
									Trt	Week (Age)	Trt × Week
Footpad dermatitis ^1^	15.4 ^d^	3.2 ^f^	47.4 ^b^	52.5 ^a^	10.8 ^e^	31.5 ^c^	33.5 ^c^	0.9	<0.001	<0.001	<0.001
Hock burns ^1^	6.5 ^c^	1.4 ^d^	12.4 ^b^	14.3 ^a,b^	3.4 ^d^	12.4 ^b^	16.1 ^a^	0.6	<0.001	<0.001	<0.001
Breast dermatitis ^1^	9.8	10.1	13.7	14.0	13.8	10.0	13.6	0.6	0.008 *	<0.001	0.001
Plumage cleanliness ^1^	13.7 ^b,c^	12.9 ^c^	18.8 ^a,b^	19.2 ^a,b^	14.7 ^a,b,c^	17.0 ^a,b,c^	19.8 ^a^	1.2	0.001	<0.001	<0.001
Gait ^1^	11.2 ^b^	8.0 ^c^	13.9 ^a^	12.8 ^a,b^	11.2 ^b^	12.7 ^a,b^	13.4 ^a,b^	0.6	<0.001	<0.001	0.004
Body weight (g)	1681.4 ^a^	1564.8 ^c^	1620.9 ^b^	1603.4 ^b,c^	1695.1 ^a^	1596.2 ^b,c^	1710.8 ^a^	13.1	0.038	<0.001	<0.001

^1^ Expressed as a tagged visual analog scale (tVAS) score between 0–100, with 0 representing the best possible outcome and 100 the worst possible outcome. Means in a row with different superscripts (^a–f^) differ significantly at *p* < 0.05. * No pairwise differences between treatment groups after Tukey-Kramer adjustment.
